# Cathétérisme veineux fémoral: cas d'un hématome fémoral Tardif

**DOI:** 10.11604/pamj.2014.17.206.3751

**Published:** 2014-03-15

**Authors:** Anouar Jarraya, Zied Triki, Jameleddine Guermazi, Wafa Abdelkafi, Michel Galinski, Abdelhamid Karoui

**Affiliations:** 1 CHU Habib Bourguiba, Sfax, Tunisie; 2 CHU Jean Verdier (APHP), Bondy, France

**Keywords:** Voie veineuse centrale fémorale, complication, hématome, anticoagulation, Femoral central venous route, complication, hematome, anticoagulation

## Abstract

La survenue d'un hématome fémoral après l'ablation d'un cathéter veineux central est une complication précoce et rare. Nous rapportons ici le cas d'un hématome fémoral survenant tardivement, 3 jours après l'ablation d'un cathéter fémoral, chez un patient de 63ans sous héparinothérapie à dose curative. Il s'agit d'un hématome volumineux mesurant 11.5 cm sur 8.2 cm sur 13 cm au niveau de la loge des adducteurs de la cuisse qui s'est compliqué d'un état de choc hémorragique. Une telle complication soulève la question d’éventuelles recommandations dans la surveillance d'une voie d'abord fémorale particulièrement chez les patients sous anticoagulation efficace.

## Introduction

Le cathétérisme veineux central est souvent nécessaire chez les patients qui vont subir des interventions chirurgicales lourdes et les patients de réanimation [1]. Toutefois, cette procédure peut conduire à des complications potentiellement mortelles d'ordre mécanique, infectieuse ou thrombotique [2]. La voie d'abord fémorale se complique plus fréquemment d'infection et de thrombose que d'hématome [3]. Nous rapportons ici le cas d'un hématome fémoral volumineux, survenant brutalement, trois jours après l'ablation du cathéter fémoral et qui s'est compliqué d'un état de choc hémorragique.

## Patient et observation

Un homme de 63 ans a été hospitalisé en chirurgie maxillo-faciale pour un carcinome épidermoïde gingivo-mandibulaire. Il avait des antécédents médicaux associant une hypertension artérielle, un diabète non insulinodépendant et une ACFA sous anti-vitamine K. Ce patient a été opéré le 14/11/2013. Il a eu une trachéotomie, une exérèse tumorale avec curage ganglionnaire en premier temps suivi par une reconstruction par un lambeau pédiculé cutanéo-musculaire du muscle grand dorsal. L'indication d'une voie veineuse centrale était posée devant le risque de variations hémodynamiques nécessitant l'administration de catécholamines. Compte tenu du type de chirurgie (chirurgie de la face), l'abord fémoral était choisi. Un cathéter double lumière Arrow 16G était mis en place en fémoral droit sans incident et la recherche d'un retour veineux était positive. Le monitorage invasif de la pression artérielle a été mis en place au niveau fémoral droit sans incidents. Les suites opératoires immédiates étaient simples avec une anticoagulation efficace par l'héparine dès la 6ème heure post opératoire relayée par l'anti-vitamine K. Cette anticoagulation était indiquée afin d’éviter la thrombose du pédicule du lambeau et vu les antécédents d'ACFA de notre patient. Lescathéters veineux et artériels fémoraux ont été ensuite enlevés sans incidents 4 jours après.

Le lambeau musculo-cutané a évolué vers la nécrose. Le patient a été repris le 05/12/2013 pour exérèse du tissu nécrosé et la reconstruction par un lambeau du muscle grand pectoral. Un cathéter triple lumière Arrow 7Fr en polyuréthane était mis en place en fémoral droit (même endroit que le précédent) sans incident alors que le cathéter artériel a été mis en radial droit. En post opératoire, ce patient a été hospitalisé en réanimation chirurgicale où le cathéter fémoral a été enlevé dès le J2 post opératoire avec une compression manuelle sur le site de ponction pendant 10 minutes. On n'a pas noté la constitution d'hématome ni un saignement anormal au niveau du site de ponction malgré qu'un traitement anticoagulant à dose curative d'héparine a été déjà en route. Le patient a été ensuite transféré au service de chirurgie maxillo-faciale.

**Figure 1:**
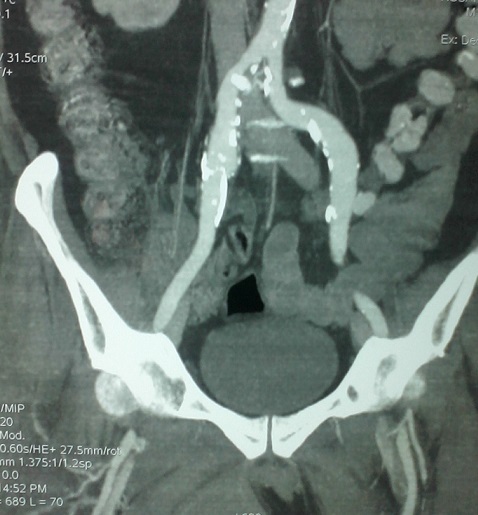
Absence d'extravasation de produits de contraste à l'angioscanner

**Figure 2:**
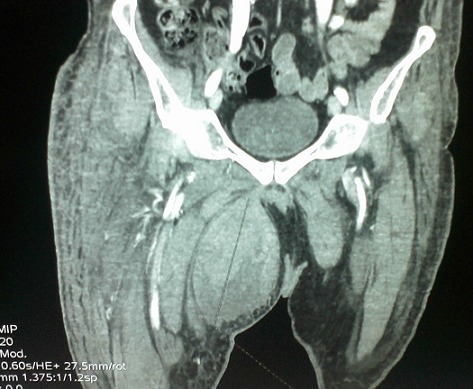
Coupe frontale de la cuisse montrant l'hématome

**Figure 3:**
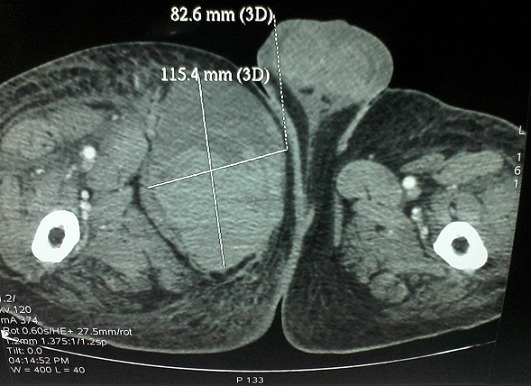
Coupe sagittale de la cuisse montrant l'hématome

**Figure 4:**
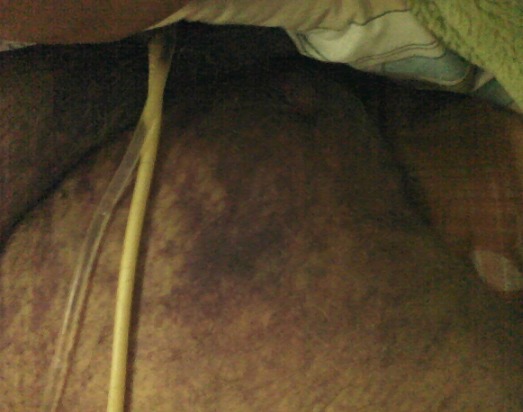
Hématome de la loge interne de la cuisse avec nécrose cutané

A J5 post opératoire, le patient a présenté une obnubilation, une tachycardie à 125 bat/min et une polypnée (FR à 30cycles / min) avec des douleurs à type d'oppression thoracique. A l'examen physique, le patient avait un hématome au niveau de la face interne de la cuisse droite d'apparition récente avec hypotension (TA: 80/45 mm Hg) et une ACFA rapide à l'ECG. La biologie a montré une déglobulisation importante avec un taux d'hémoglobine à 3.9 g/dl et une élévation des troponines Ic à 0.46ng/ml. Le bilan d'hémostase était perturbé avec un taux de prothrombine à 58% et un TCK ratio à 2.41. Un scanner thoraco-abdomino-pelvien a été pratiqué en urgence et a montré l'absence d’épanchement thoracique ou abdominal. Par contre, aucune extravasation du produit de contraste n'a été objectivé (Figure 1). L'hématome était situé au niveau de la loge interne de la cuisse droite refoulant les adducteurs et mesurant 13/8.2/11.5 cm (Figure 2, Figure 3)Le patient a été transféré en milieu de réanimation où il a eu une transfusion de 04 CGR et 04 PFC afin de corriger la chute d'hémoglobine et les troubles de l'hémostase avec arrêt de l'héparinothérapie. Il était alors décidé, après avis chirurgical, d'une surveillance médicale sans indication opératoire. L’évolution a été marquée par amélioration de l’état hémodynamique, de l'anémie (Hb à 8.9 g/dl), de l'hémostase (TP = 84%, TCA ratio =1.01) et diminution des troponines Ic. Quelques heures après, des signes de souffrance cutanée en regard de l'hématome ont imposé son évacuation chirurgicale (Figure 4). L’évolution était cliniquement favorable en quelques jours.

## Discussion

La formation d'un hématome suite au cathétérisme de la veine fémorale est une complication qui peut se voir au moment de la ponction surtout en cas de ponction accidentelle de l'artèrefémorale [4]. Des cas isolés d’épanchement intrapéritonéal iatrogène suite à l'insertion d'un cathéter veineux fémoral ont été décrits dans la littérature [5, 6]. Cependant, la survenue d'un hématome trois jours après l'ablation d'un cathéter veineux fémoral est une complication exceptionnelle. Dans notre cas, ce saignement pourrait être favorisé par le traitement anticoagulant à dose curative et la fragilisation de la veine qui a été cathétérisée deux fois successives. En plus, le cathéter en polyuréthane peut à son tour être incriminé car il est moins flexible et favorise les perforations secondaires [7]. Un diamètre large (14G) ou un bilumière augmentent de façon significative le risque de complications hémorragiques [8]. Cependant l'hémorragie par perforation secondaire dans le territoire cave inférieur reste exceptionnelle [9]. Le cathéter veineux fémoral peut se compliquer tardivement d'infection et de thrombose plutôt que de saignement [3]. Il existe des cas rapportés dans la littérature qui ont décrit des complications habituellement précoces mais qui ont été vu tardivement [10].

Il est légitime de s'interroger si on aurait pu éviter cet incident [11]. En fait, une indication raisonnée de cathétérisme veineux central reste la solution essentielle pour diminuer la fréquence des complications [1]. Outre, l'intérêt de l’écho guidage permet d’éviter les traumatismes des structures anatomiques adjacentes ce qui n'est pas le cas pour ce patient [12]. En plus, la compression manuelle de la veine fémorale pendant 10 minutes chez un patient sous anticoagulation curative pourrait être insuffisante et engendrer un saignement à distance. Une surveillance clinique de l’état local du point de ponction après ablation du cathéter permettrait de détecter précocement la formation progressive d'une collection surtout si le patient est sous anticoagulant [3, 11]. Cette observation permet en tout cas de poser le problème de de la surveillance des cathéters centraux dans notre hôpital: il n'est retrouvé aucune traçabilité de la vérification quotidienne, voire pluriquotidienne de la région fémorale dans le dossier médical ou infirmier. Elle est le point de départ d'une démarche d’évaluation de nos pratiques professionnelles.

## Conclusion

La formation d'un hématome fémoral survenant brutalement 3 jours après l'ablation d'un cathéter fémoral n'a jamais été rapportée. Il est paru opportun de la signaler. Cette observation permet en tout cas de poser le problème de la surveillance des cathéters centraux surtout en cas d'anticoagulation efficace. La surveillance après l'ablation du cathéter doit être encadrée des mêmes mesures de contrôleque pour un cathéter en place.
